# Antiviral Activity of Benzoic Acid Derivative NC-5 Against Influenza A Virus and Its Neuraminidase Inhibition

**DOI:** 10.3390/ijms20246261

**Published:** 2019-12-12

**Authors:** Min Guo, Jiawei Ni, Jie Yu, Jing Jin, Lingman Ma, Huixing Zhang, Dechuan Wang, Xue Zhang, Jie Dou, Changlin Zhou

**Affiliations:** 1 State Key Laboratory of Natural Medicines, School of Life Science and Technology, China Pharmaceutical University, 24 Tong Jia Xiang, Nanjing 210009, China; gm478220352@126.com (M.G.); jiawei_7072@163.com (J.N.); yujie_111222@163.com (J.Y.); cpubiojj@163.com (J.J.); malingman1987@126.com (L.M.); zhangxuezhangfan@163.com (X.Z.); 2 Department of Organic Chemistry, School of Science, China Pharmaceutical University, 24 Tong Jia Xiang, Nanjing 210009, China; zhanghuixing_111@163.com (H.Z.); wdc@cpu.edu.cn (D.W.)

**Keywords:** benzoic acid derivatives, antiviral activity, neuraminidase inhibition, oseltamivir-resistant

## Abstract

The currently available drugs against influenza A virus primarily target neuraminidase (NA) or the matrix protein 2 (M2) ion channel. The emergence of drug-resistant viruses requires the development of new antiviral chemicals. Our study applied a cell-based approach to evaluate the antiviral activity of a series of newly synthesized benzoic acid derivatives, and 4-(2,2-Bis(hydroxymethyl)-5-oxopyrrolidin-l-yl)-3-(5-cyclohexyl-4H-1,2,4-triazol-3-yl)amino). benzoic acid, termed NC-5, was found to possess antiviral activity. NC-5 inhibited influenza A viruses A/FM/1/47 (H1N1), A/Beijing/32/92 (H3N2) and oseltamivir-resistant mutant A/FM/1/47-H275Y (H1N1-H275Y) in a dose-dependent manner. The 50% effective concentrations (EC_50_) for H1N1 and H1N1-H275Y were 33.6 μM and 32.8 μM, respectively, which showed that NC-5 had a great advantage over oseltamivir in drug-resistant virus infections. The 50% cytotoxic concentration (CC_50_) of NC-5 was greater than 640 μM. Orally administered NC-5 protected mice infected with H1N1 and H1N1-H275Y, conferring 80% and 60% survival at 100 mg/kg/d, reducing body weight loss, and alleviating virus-induced lung injury. NC-5 could suppress NP and M1 protein expression levels during the late stages of viral biosynthesis and inhibit NA activity, which may influence virus release. Our study proved that NC-5 has potent anti-influenza activity in vivo and in vitro, meaning that it could be regarded as a promising drug candidate to treat infection with influenza viruses, including oseltamivir-resistant viruses.

## 1. Introduction

The influenza A virus belongs to the *Orthomyxoviridae* family and is a major cause of severe epidemics of respiratory illness [1]. The genome of the influenza virus contains eight segmented and negative-stranded RNAs, encoding for eleven proteins: hemagglutinin (HA), neuraminidase (NA), nucleoprotein (NP), Non-structural protein 1 (NS1), NS2, polymerase acidic protein (PA), Matrix protein 1 (M1), M2, polymerase basic 1 (PB1) and PB2, PB1-F2. Neuraminidase is on the surface of the envelope; its function is to cleave the sialic acid residues that attach the progeny virus to infected cells, thereby detaching the progeny virus and completing the cycle of virus infection and propagation [2]. The NA and M1 proteins have proven to be effective targets for anti-influenza viral therapy [3]. Influenza NA is a homotetramer classified into two phylogenetically distinct groups; compared to group two (N2, N3, N6, N7 and N9), group one (N1, N4, N5 and N8) has an “150-cavity” near the active area [4]. The “150-cavity” is a loop of amino acids adopting an open conformation, consisting of residues 147–152 together with the active site residues Asp151 and Glu119 [5]. Benefitting from alkylation and guanidylation of the oseltamivir C-5 amino acid and the same transformations at position C-4 of zanamivir, the two molecules target the “150-cavity” of the NA protein, inhibiting its enzymatic activity and preventing the tethered progeny virus from escaping from host cells [6,7]. However, due to the frequent emergence of drug-resistant influenza viruses, the usage of these drugs has been greatly limited [8,9,10], making the discovery of novel anti-influenza drugs an even more urgent task.

**Figure 1:**
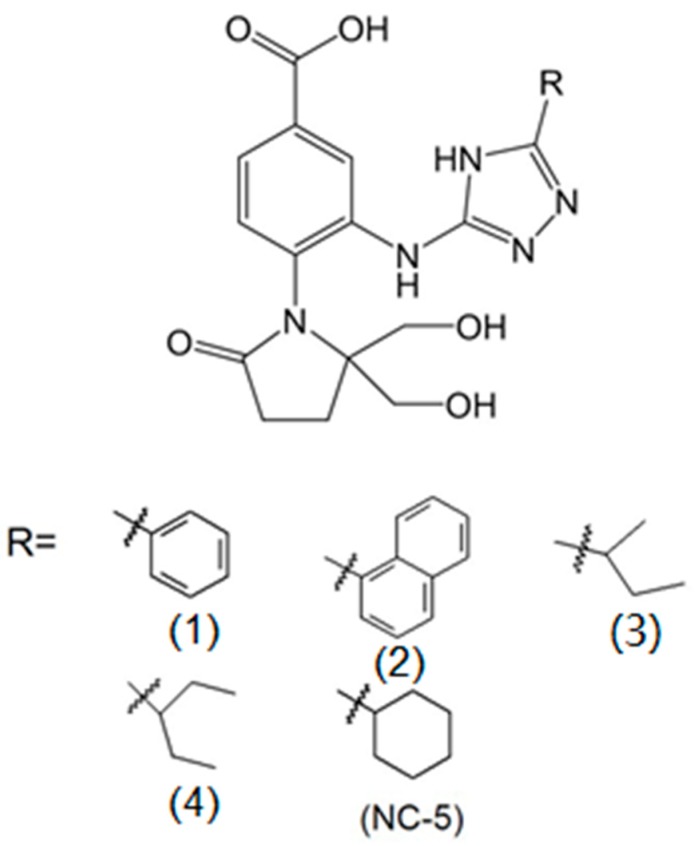
Chemical structure of newly synthesized benzoic acid derivatives. R=: substituent group on the triazole; R1: phenyl R2: naphthaleneyl R3: sec-butyl R4: pentan-3-yl R5: cyclohexyl. NC-5: 4-(2, 2-Bis (hydroxymethyl)-5-oxopyrrolidin-l-yl)-3-(5-cyclohexyl-4H-1, 2, 4-triazol-3-yl)amino) benzoic acid.

Benzoic acid derivatives have been reported to possess anti-influenza virus activities. Among them, BANA-206, the first achiral molecule, was reported to show sub-micromolar antiviral potency against the influenza A virus [11,12]. Some compounds have been successfully designed by the conjugation method, including compounds BTA938 [13] and ZA-7-CA [14]; their anti-influenza activity was enhanced. Based on combination principles as well as the principle of functional groups, we integrated triazole into BANA-206 on the C3 side chain and designed a series of benzoic acid derivatives to obtain potential influenza virus inhibitors with improved antiviral activity. In our research, five compounds (Figure 1) were evaluated for their antiviral activities in infected-cell models. Ultimately, 4-(2, 2-Bis (hydroxymethyl)-5-oxopyrrolidin-l-yl)-3-(5-cyclohexyl-4H-1, 2, 4-triazol-3-yl) amino) benzoic acid, termed NC-5, emerged as the most effective compound. We tested its antiviral activity against A/FM/1/47 (H1N1), A/Beijing/32/92 (H3N2) and A/FM/1/47-H275Y (H1N1-H275Y) in vitro and against H1N1 and H1N1-H275Y in vivo. The mechanistic studies indicated that NC-5 may cause the virus to be unable to escape from its host cells through inhibiting NA activity.

## 2. Results

### 2.1. The Antiviral Activities of NC-5 and its Analogs against Influenza Virus A/FM/1/47 (H1N1)

BANA-206, a benzoic acid derivative, was reported to show potent antiviral activity [15]. The analogs of oseltamivir and zanamivir that possess a triazole substituent had been reported to inhibit the influenza virus via insertion into the 150-cavity near the active site of NA. These results prompted us to consider the use of triazole substituents to increase the NA-inhibitory effect. The cytopathic effect (CPE) reduction assay was utilized to screen and identify compounds that displayed a reduction in the CPE on the influenza H1N1 virus at 10 μM and 100 μM (Table 1). We found that the derivatives possessed antiviral activity ranging from 7.3% to 16.7% at 10 μM, and 7.8% to 83.7% at 100 μM. The data in Table 1 shows that all of the compounds exhibited a low viral inhibitory effect at 10 μM. Compounds bearing a saturated alicyclic hydrocarbon (Compound 5) showed a better effect than compounds with phenyl substituents (Compounds 1 and 2) or chain substituents (Compounds 3 and 4). Substituents consisting of large groups with saturated rings might be advantageous options for designing antiviral compounds because of the possibility of the triazole group fitting into the 150-cavity in NA. Compound 5, a diethyl triazole benzoic acid derivative named 4-(2,2-bis(hydroxymethyl)-5-oxopyrrolidin-l-yl)-3-(5-cyclohexyl-4H-1,2,4-triazol-3-yl)amino) benzoic acid, significantly reduced the viral CPE in Madin–Darby canine kidney (MDCK) cells with an inhibition rate of 83.7% at 100 μM, the highest inhibition rate among the derivatives.

### 2.2. NC-5 has the Ability to Inhibit Neuraminidase Activity

**Figure 2:**
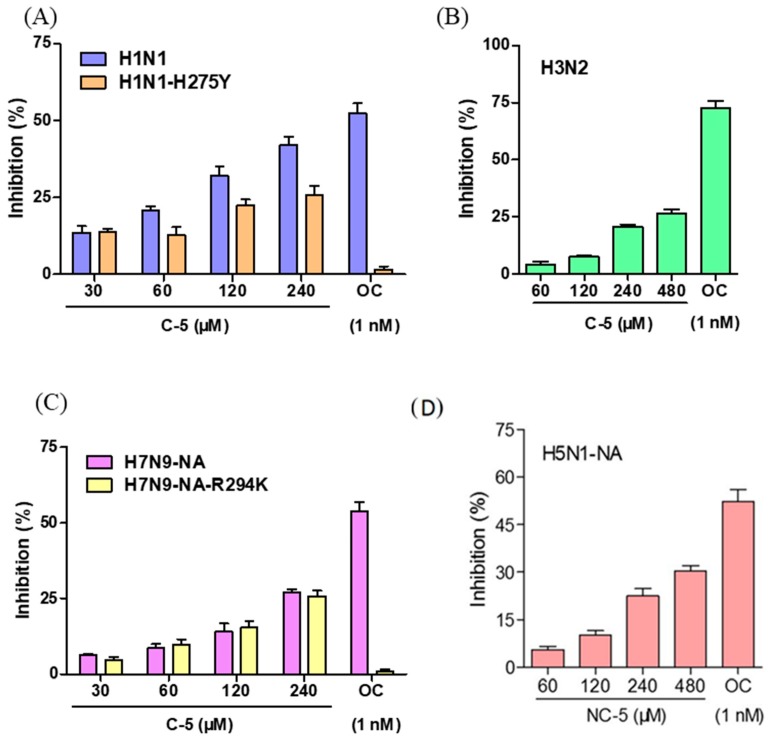
The inhibitory activity of NC-5 on influenza A virus neuraminidase (NA). (**A**) NA-inhibitory effect on A/FM/1/47 (H1N1) and A/FM/1/47-H275Y (H1N1-H275Y). (**B**) A/Beijing/32/92 (H3N2). (**C**) A/Anhui/1/2013 (H7N9) and A/Anhui/1/2013-R294K (H7N9-R294K). (**D**) A/Vietnam/1203/2004 (H5N1). Oseltamivir carboxylate (OC) (1 nM) was used as a positive control. The results are represented as the mean ± S.D. of three independent experiments, *p <0.05, **p <0.01, ***p <0.001, compared with the dimethyl sulfoxide (DMSO) control.

In the NA activity inhibition assay, the NA activities of wild-type H1N1 (Figure 2A) and H3N2 (Figure 2B) as well as the CHO-K1 expression of NA proteins of highly pathogenic avian influenza viruses H5N1 (Figure 2D) and H7N9 (Figure 2C) were suppressed by NC-5. The NA activities of oseltamivir-resistant viruses H1N1-H275Y (Figure 2A) and H7N9-R294K (Figure 2C) were also inhibited by NC-5 with a weaker or similar inhibition effect. The inhibition rate of NC-5 at 480 μM on all NAs was above 25% and the IC50 of each strain was higher than 480 μM. For oseltamivir-resistant strains, the inhibition rates of oseltamivir at 1 nM were less than 2% compared with inhibition rates of approximately 50% toward wild-type strains. NC-5 suppressed the NA activity of H1N1-H275Y and H7N9-R294K and the inhibition rates were both 25.8% at 480 μM. The effectiveness of NC-5 on wild-type and oseltamivir-resistant virus strains indicated that NC-5 might be a mild NA inhibitor that could be employed in the treatment of drug-resistant viral infections.

### 2.3. NC-5 inhibited the Replication of Influenza Viruses A/FM/1/47 (H1N1), A/FM/1/47-H275Y (H1N1-H275Y) and A/Beijing/32/92 (H3N2) in Cell Culture

**Figure 3:**
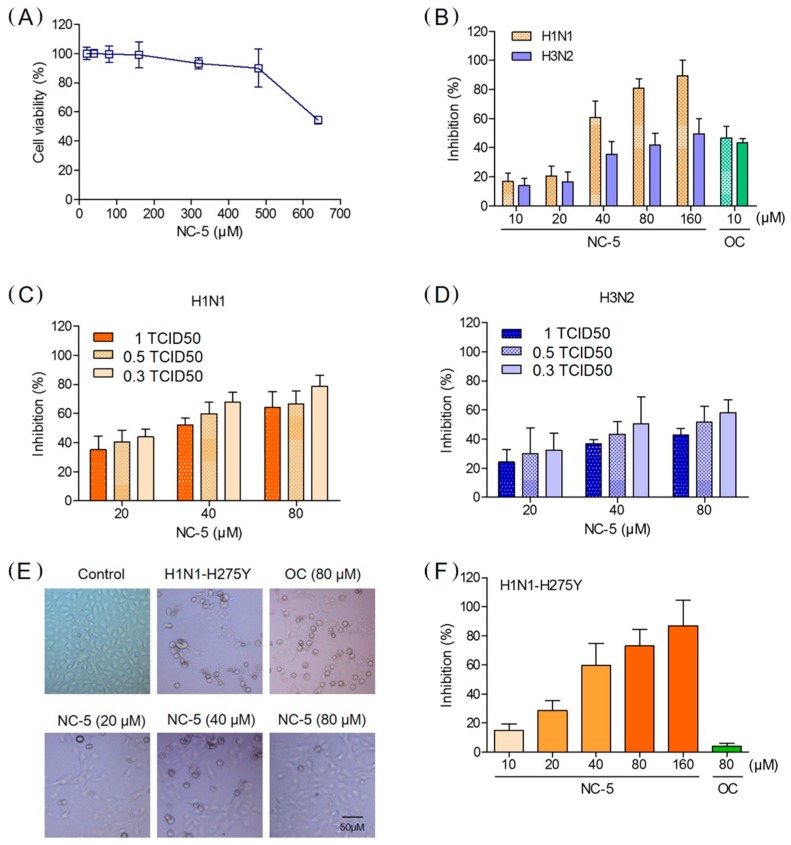
Inhibitory effect of NC-5 on wild-type and oseltamivir-resistant influenza A virus in Madin–Darby canine kidney (MDCK) cells. (**A**) Cellular toxicity of NC-5 in MDCK cells. (**B**) The inhibition rate of NC-5 (10–160 μM) on influenza A virus A/FM/1/47 (H1N1) and A/Beijing/32/92 (H3N2). MDCK cells were infected with 100 TCID50 of A/FM/1/47 (H1N1) and A/Beijing/32/92 (H3N2). Oseltamivir carboxylate (OC) (10 μM) was used as a positive control. (**C**) The inhibition rate of NC-5 (20–80 μM) against influenza A virus H1N1 in MDCK cells at different TCID50. (**D**) The inhibition rate of NC-5 (20–80 μM) against influenza A virus H3N2 in MDCK cells at different TCID50. (**E**) Microscopic image for A/FM/1/47-H275Y (H1N1-H275Y) virus-infected NC-5 (20–80 μM) treated MDCK cells at 48 h post-infection. MDCK cells were infected with 100 TCID50 of H1N1-H275Y. DMSO was used as a vehicle control. OC (80 μM) was also used as a control. (**F**) Inhibition rate of NC-5 (10–160 μM) on H1N1-H275Y virus infected MDCK cells. OC (80 μM) was used as a control. The results are presented as the mean ± S.D. of three independent experiments.

Based on the results of the CPE reduction assay (Table 1), we tested other influenza virus strains including an oseltamivir-resistant strain to confirm the results above. In the additional CPE reduction experiments, NC-5 exhibited a considerable protective effect. H1N1, H1N1-H275Y and H3N2 were tested with oseltamivir carboxylate (OC) as a control. NC-5 (10–160 μM) showed significant dose-dependent anti-influenza activity. The inhibition rates at 80 μM against H1N1 and H3N2 were 81.1% and 42.1% (Figure 3B), respectively, with minimal adverse effects on the host cell at high concentrations (Figure 3A). The effects were also confirmed when cells were infected with different viruses with a tissue culture infective dose 50 (TCID50)of 1, 0.5, and 0.3 (Figure 3C,D). As in the case of H1N1-H275Y, the inhibition rate of NC-5 at 80 μM was 72.9%, while that of OC was only 4.0% (Figure 3F). The reduction in CPE caused by H1N1-H275Y was confirmed by direct microscopic observation, which detected a far lower CPE than the dimethyl sulfoxide (DMSO) control and the OC-treated cells (Figure 3E). NC-5 at 20–80 μM was able to protect cells from necrobiosis and detachment, while OC at 80 μM showed little inhibitory activity.

**Figure 4:**
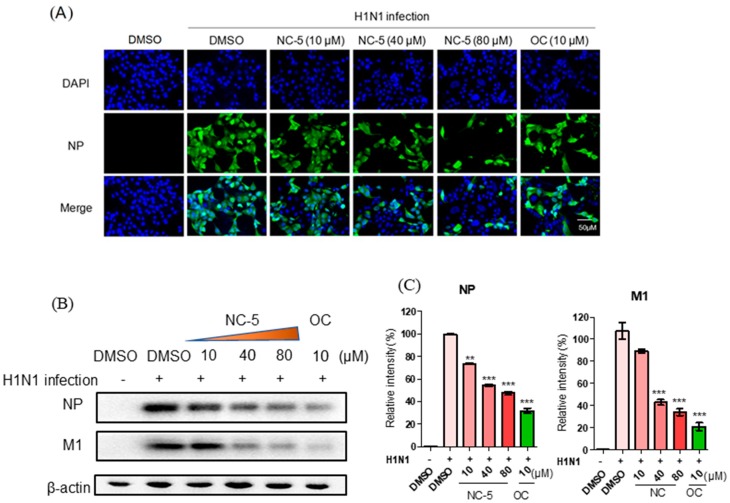
NC-5 suppression of A/FM/1/47 (H1N1) influenza viral replication and its effect on NP and M1 protein synthesis. (**A**) Fluorescence microscopy images of NC-5-treated (10–80 μM) H1N1-infected MDCK cells with 1.5 × 10^3^ TCID50 at 24 h post-infection. Oseltamivir carboxylate (OC) (10 μM) was used as a positive control. (**B**) MDCK cells were infected with 1.5 × 10^3^ TCID50 of H1N1, treated with NC-5 (10–80 μM) or OC (10 μM) and administered 2 h post-infection (p.i.). Cells at 24 h p.i. were harvested and subjected to Western blotting with NP and M1 antibodies. (**C**) The results of the Western blot in (**B**) were analyzed using ImageJ software. The expression of NP and M1 proteins was quantified. All results are presented as the mean ± S.D. of three independent experiments, ** *p* <0.01, *** *p* <0.001 compared with the DMSO-treated group.

The results of the Western blot analysis, conducted to investigate the influence of NC-5 on H1N1 virus NP and M1 protein level, showed that NC-5 could significantly decrease the expression of NP and M1 protein (Figure 4B,C) 48 h post-infection in a dose-dependent manner, indicating that NC-5 inhibits influenza virus infection by interfering with viral replication. This result was also supported by the fluorescence microscopy observations of the NP distribution in the host cell (Figure 4A).

These results indicate that NC-5 effectively inhibits both wild-type and oseltamivir-resistant mutant influenza viruses in vitro and could reduce the level of NP and M1 viral proteins, suppressing viral replication.

### 2.4. Effective Protection by NC-5 in Mice Infected with Influenza Virus A/FM/1/47 (H1N1) and Oseltamivir-Resistant Mutant A/FM/1/47 (H1N1-H275Y)

**Figure 5:**
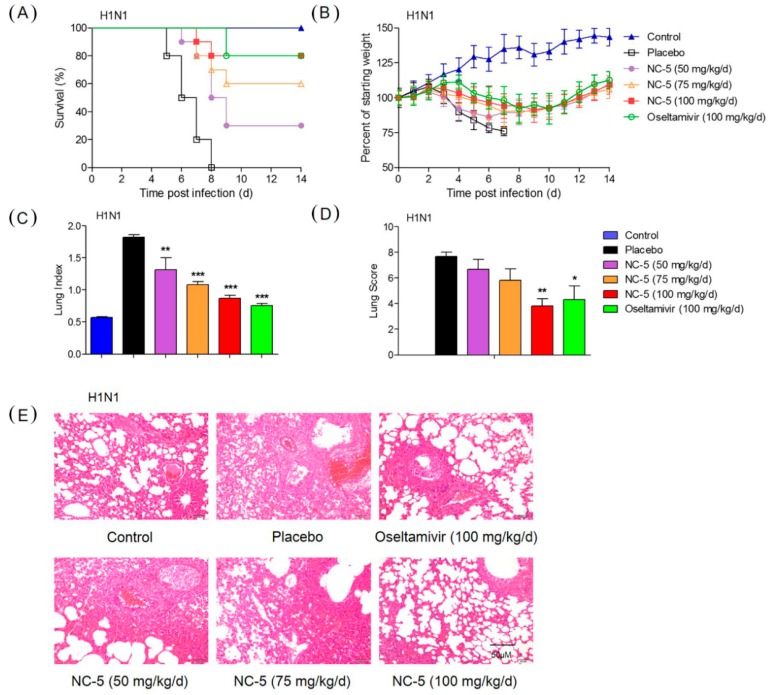
Protective effect of NC-5 on mice infected with A/FM/1/47 (H1N1) virus. The ICR mice were infected with influenza A virus (8 × LD_50_), and ten mice in each group received placebo, NC-5 or oseltamivir phosphate (OS) twice daily for 5 days. (**A**) Survival rate. (**B**) Body weight. On day 5 p.i., five mice per group were sacrificed for lung pathology examinations. (**C**) Lung index. (**D**) Histological lung injury scores of mice in each group. (**E**) H & E staining of lung tissues (*n* = 5). Data are presented as the mean ± S.D. of three independent experiments, * *p* <0.05, ** *p* <0.01, *** *p* <0.001 compared with the placebo group.

**Figure 6:**
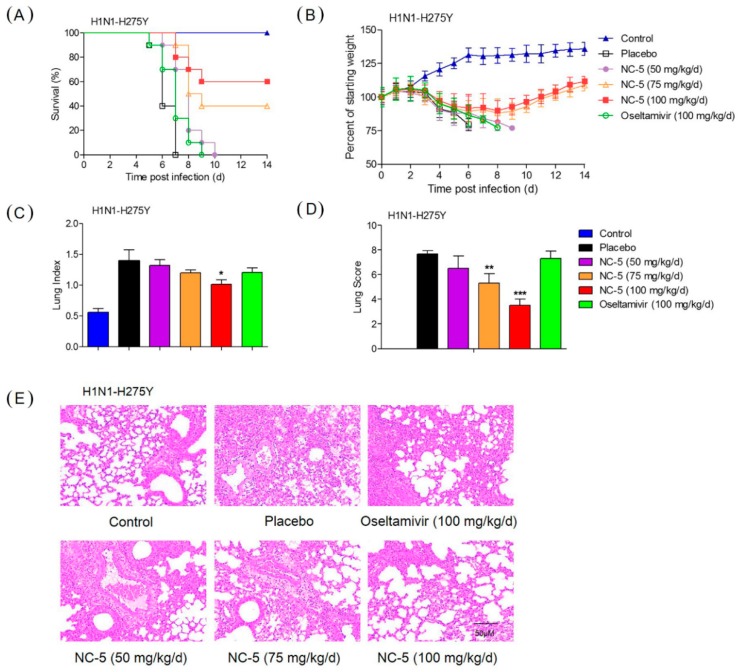
Therapeutic effect of NC-5 on mice infected with the oseltamivir-resistant influenza A virus A/FM/1/47-H275Y (H1N1-H275Y). The ICR mice were intranasally infected with H1N1-H275Y virus (8 × LD50), and ten mice in each group received a placebo, NC-5 or oseltamivir phosphate (OS) twice daily for 5 days. (**A**) Survival rate. (**B**) Body weight monitored daily. On day 5 p.i., five mice per group were euthanized for lung pathology examination and lung index calculation. (**C**) Lung indices. (**D**) Histological scores (*n* = 6). (**E**) H & E staining of sectioned lungs. Data are represented as the mean ± S.D. of three independent experiments, * *p* <0.05, ** *p* <0.01, *** *p* <0.001 compared with the placebo group.

A dose-response study in a virus-infected murine model was designed to evaluate the efficacy of NC-5 against the influenza virus in vivo. Body weight, survival and clinical manifestations were recorded for 14 days. The mice infected with wild-type and oseltamivir-resistant viruses presented with severe clinical manifestations, including a ruffled coat, inappetence, inactivity, shudder and body weight loss three days after infection. Among mice infected with H1N1, those given saline died on day eight, while mice being given NC-5 (100 mg/kg/d) or OS (100 mg/kg/d) both had a survival rate of 80% (Figure 5A). In the case of H1N1-H275Y, the mice given OS all died on the ninth day, while NC-5 exhibited significant protection effect with 75 mg/kg/d and 100 mg/kg/d NC-5, increasing survival to 40% and 60% (Figure 6A), respectively. The clinical signs and body weight of the surviving mice were recovered by day eight, although the mouse body weights were still lower than in the normal group (Figure 5B and Figure 6B).

On the fifth day post-infection, five mice in each group were sacrificed for lung pathology examination. NC-5 treatment also led to an obvious reduction in influenza-induced lung pathology. As shown in Figure 5E and Figure 6E, severe hyperemia appeared in the alveolar walls, and the spaces between the alveolar walls were filled with moderate inflammatory infiltrates of neutrophils, macrophages and lymphocytes in the H1N1 and H1N1-H275Y virus-infected models as well as in mice given OS following infection with H1N1-H275Y. The lungs of mice given NC-5 exhibited reduced histopathological symptomatic changes in a dose-dependent manner. The lung parameters showed that treatment with NC-5 could significantly decrease the lung index and lung injury score (Figure 5C,D) of the H1N1 infected mice, exhibiting a significant effect at a dosage of 100 mg/kg/d (lung index: 0.9, lung injury score: 3.8) compared with the model group (lung index: 1.8, lung injury score: 7.7). The lung parameters of mice infected with H1N1-H275Y and the mice administered OS (Figure 6C,D, lung index: 1.2, lung injury score: 7.3) were similar to those of the corresponding models (lung index: 1.4, lung injury score: 7.7), while the groups that received NC-5 at 100 mg/kg/d showed a significant improvement in condition (lung index: 1.0, lung injury score: 3.5). The results above and other parameters indicate that NC-5 could prolong survival, release pathological changes in the lungs and reduce body weight loss. NC-5 demonstrates potent therapeutic efficacy in vivo against wild-type and oseltamivir-resistant influenza viruses.

### 2.5. NC-5 Suppressed Influenza Virus A/FM/1/47 (H1N1) in the Late Stages of its Life Cycle by Interfering NP and M1 Protein Expression but Showed no Effect on RNA Polymerase

**Figure 7:**
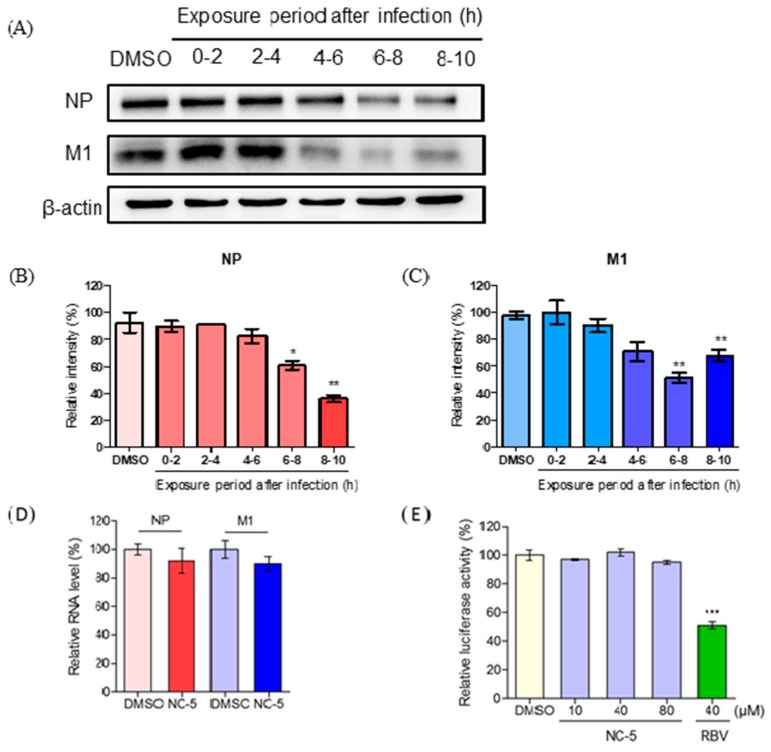
NC-5 suppressed influenza A virus replication by inhibiting NP and M1 protein expression without affecting mRNA transcription of NP and M1 or RNA polymerase. (**A**) MDCK cells were infected with H1N1 at a multiplicity of infection (MOI) of 5, and NC-5 was given at intervals of 0–2 h, 2–4 h, 4–6 h, 6–8 h, and 8–10 h post-infection. The cell lysates were harvested at 10 h and subjected to Western blot analysis. The results were analyzed with ImageJ software. (**B**) Relative amounts of NP protein calculated in comparison to the DMSO control. (**C**) Relative amounts of M1 protein calculated in comparison to the DMSO control. (**D**) Relative RNA levels of the NP and M1 genes determined in a real-time qPCR experiment. The experiment was performed in triplicate and normalized to Glyceraldehyde 3-phosphate Dehydrogenase (GAPDH), relative to the DMSO control. (**E**) The relative luciferase activity of 293T cells transfected with plasmids containing PB1, PB2, PA, NP, influenza virus-like RNA encoding firefly luciferase (vNS-Luc) and pCMV β-gal for 24 h. Cell lysates were used for the luciferase assay. The activities were normalized, and the relative luciferase activity was calculated to the DMSO control. The results are represented as the mean ± S.D. of three independent experiments, * *p* <0.05, ** *p* <0.01, *** *p* <0.001 compared with the DMSO control.

We tried to explore the potential mechanisms by which NC-5 inhibits influenza infection. The complete life cycle of the influenza virus is approximately 8–10 h and it is divided into the following three stages: virus entry (0–2 h), viral genome replication and translation (2–8 h), and progeny virion release (8–10 h) [16]. A time-of-addition experiment was performed via adding NC-5 to influenza-infected cells at different stages of the virus replication cycle and then measuring the relative level of NP and M1 proteins 10 h later. The cells were co-incubated with the virus at 4 °C for 1 h, then, after washing with phosphate-buffered saline (PBS) twice, the cells were cultured at 37 °C. Two milliliters of medium was added to each well and NC-5 (80 μM) was added at 0–2 h, 2–4 h, 4–6 h, 6–8 h, and 8–10 h post-infection. When NC-5 was added during the replication stage and release stage (4–6 h or 6–8 h after infection), the M1 protein level was significantly reduced with an inhibition rate of 48.2% in 6–8 h (Figure 7A,C). NP protein expression showed a slight decrease at the late biosynthesis stage (Figure 7A,B). However, in the adsorption stage or the early biosynthesis stage (0–2 h or 2–4 h after infection), no inhibitory activity was observed. The results suggest that NC-5 may disrupt RNA polymerase-mediated transcription and replication as well as the virus assembly and release stages. However, the results of the luciferase experiments did not indicate inhibition of RNA polymerase compared with the DMSO control (Figure 7E). The NP and M1 RNA levels at 6 h post-infection (Figure 7D) had not decreased much in the qPCR analysis results. Thus, NC-5 did not interrupt the transcription of NP and M1.

## 3. Discussion

Here we report a diethyl triazole benzoic acid derivative, NC-5, that displays anti-influenza virus activity in vitro and in vivo. The influenza virus produces clear and rapid CPE following cell death in vitro [17]. The compound exhibited relatively broad-spectrum activity among the wild-type and oseltamivir-resistant virus strains with limited cytotoxicity. NC-5 effectively alleviated the CPE and inhibited the replication of H1N1 and H1N1-H275Y viruses with 50% effective concentration (EC_50_) values of 33.6 μM and 32.8 μΜ. However, for influenza virus H3N2, the EC_50_ value was greater than 160 μM, possibly because the inelastic 150-loop differs from that in N1 [18]. This difference may make it difficult for triazole substituents to insert into the 150-cavity in NA, which is of great importance in virulence. The oral administration of NC-5 significantly enhanced the survival rates of wild-type- and oseltamivir-resistant virus-infected mice, as 80% and 60% of mice were protected from death, respectively, at 100 mg/kg/d. However, OS failed to protect mice infected with H1N1-H275Y virus, and all of the mice died on day nine post-infection. Lung injury and all other parameters also confirmed the remarkable therapeutic effects of NC-5. Together, the in vitro and in vivo results suggest that NC-5 exhibits outstanding antiviral activity. The compound reduces viral replication, protein synthesis and CPE in cell culture, and it suppresses viral propagation and alleviates associated severe symptoms caused by viral infections in mice. All the data demonstrate that NC-5 possesses potent activity against influenza viruses, including oseltamivir-resistant viruses.

Nucleoprotein (NP) and matrix protein 1 (M1) are structural proteins that are essential for the integrity of a virus [19]. Both can reflect the expression state of virus replication. In experiments exploring the mechanism of antiviral activity, we found that the activity against influenza virus took effect in the biosynthesis stage with the protein synthesis of both NP and M1. NC-5 slightly decreased the NP and M1 RNA levels. However, it did not affect RNA polymerase activity and NC-5 did not interfere with the entry of the virus into the host cells but rather played an important role in the virus release stage due to its NA inhibitory activity, which was effective against all strains and NA proteins employed. The experimental results showed that NC-5 has potential as a new candidate NA inhibitor. We hypothesize that NC-5 may affect the post-transcriptional regulation that functions in the viral protein synthesis process which we may explore in subsequent studies.

Compared to the two widely used anti-influenza drugs, zanamivir and oseltamivir, BANA-206 and NC-5 are achiral molecules exhibiting great potent anti-influenza activity, which can avoid chemical synthesis difficulty and high production cost. The Molecular Operating Environment(MOE) software was used for the molecular simulation docking of NC-5 and NA, and the docking simulation results showed that the benzene ring of NC-5 was conjugated with Arg118, two hydroxyls at pyrrolidone hydrogen-bonded with Tyr347, and Arg371 in H1N1-NA, respectively. It has been reported that the resistance mutations of influenza virus H1N1 mostly occur at His275 and Arg292 sites [20], different from the active amino acid sites which interact with NC-5, which makes NC-5 a good potential drug for the treatment of drug-resistant influenza. However, to corroborate the docking simulation results, further experiments should be designed to investigate the exact amino acids of the viral NA that interact with NC-5.

## 4. Materials and Methods

### 4.1. Cells, Viruses and Plasmids

Cell culture: MDCK cells and human embryonic kidney (HEK) 293T cells were maintained in Dulbecco’s modified Eagle’s medium (DMEM) (Invitrogen) supplemented with 10% fetal bovine serum (FBS) (Invitrogen) at 37 °C in a 5% CO_2_ incubator. Chinese hamster ovary (CHO-K1) cells were grown in DMEM/F12 medium supplemented with 10% FBS.

Virus production: The influenza A virus used in this study, containing A/FM/1/47 (H1N1), A/Beijing/32/92 (H3N2) and the H1N1 oseltamivir-resistant mutant strain A/FM/1/47-H275Y (H1N1-H275Y) (by introducing a histidine-to-tyrosine substitution at position 275 into NA [21]), were propagated in 10-day-old embryonated chicken eggs. Viruses were stored at −80 °C.

Plasmid construction: The recombinant plasmids pcDNA3.1(+), which can stably express NA protein sequences of A/Vietnam/1203/2004 (H5N1), A/Anhui/1/2013 (H7N9) and A/Anhui/1/2013-R294K (H7N9-R294K) were transfected into CHO-K1 cells using Lipofectamine^®^ 2000 Reagent (Invitrogen™, Carlsbad, CA, USA).

Expression plasmids for influenza A virus A/WSN/33 (H1N1) proteins (PB1, PB2, PA and NP), an influenza virus-like RNA encoding firefly luciferase (vNS-Luc), and pCMV β-gal plasmid [22] were kindly provided by Xin Ye, Institute of Microbiology, Chinese Academy of Sciences.

### 4.2. Compounds

Oseltamivir phosphate (OS) and oseltamivir carboxylate (OC) were purchased from MedChem Express (Shanghai, China). The test compounds were synthesized and provided by Dechuan Wang at the Department of Organic Chemistry, China Pharmaceutical University. Its synthesis process was similar to SA-2, which was synthesized according to published procedures [21,23]. The structures and purity of all synthesized compounds were verified by 1H NMR, and the purity was all >95%. Each compound was dissolved in DMSO as a 50 mM stock solution.

### 4.3. Determination of Influenza a Virus TCID50

The influenza A virus was serially diluted and added to specific wells, which contained 5 × 10^3^ MDCK cells/well. Infected cells were incubated at 37 °C for 48 h, after which 10 μL of Cell Counting Kit-8 (CCK-8, Dojindo, Japan) reagent [24] was added to each well. After incubation for 2 h, the absorbance at 450 nm was detected on a plate reader. The TCID50 was determined using the Reed–Müench method.

### 4.4. Cytopathic Effect (CPE) Reduction Assay

MDCK cells were cultured in 96-well microplates at a density of 5 × 10^3^ cells/well for 24 h and infected with 100 TCID50 H1N1, H1N1-H275Y or H3N2 virus for 2 h. The cells were cultured in maintenance medium (DMEM containing 2 μg/mL TPCK-trypsin) and treated with 0.2% DMSO (a vehicle to dissolve test compounds) or different concentrations of test chemicals. Cell viability was measured with CCK-8 reagent as previously mentioned after 48 h of incubation. The EC_50_ values were calculated by determining the concentrations of the compounds needed to inhibit 50% CPE against the compound concentrations. GraphPad Prism 5 was used as fitting software. The 50% cytotoxic concentration (CC_50_) against MDCK cells was determined by procedures similar to those for EC_50_ determination without virus infection. The inhibition rate was calculated using the following equation [25]: inhibition rate (%) = [(A − B)/ (C − B)] × 100, where A: mean optical density of test, B: mean optical density of virus controls, C: mean optical density of cell controls.

### 4.5. Antiviral Activity of NC-5 in vivo

Male ICR mice (6 weeks old) were supplied by the Model Animal Center of Yangzhou University (Yangzhou, Jiangsu, China) and kept in the animal center at China Pharmaceutical University. Experiments were performed as previously described [26]. NC-5 was first dissolved in DMSO and then mixed with 0.5% sodium carboxymethylcellulose (CMC-Na) solution for in vitro experiments; the final concentration of DMSO was 5% as previously reported [21]. The ICR mice were divided into a control group (mice without viral infection), a placebo group (infected mice without treatment), an NC-5 treated group (50 mg/kg/d, 75 mg/kg/d, and 100 mg/kg/d), and an oseltamivir-treated group (100 mg/kg/d), each group consisting of 10 mice. Mice were anesthetized by inhalation of diethyl ether and then intranasally infected with 50 μL of a suspension of 8 × LD50 of the mouse-adapted influenza A/FM1/1/47 (H1N1), A/FM/1/47-H275Y virus diluted in phosphate-buffered saline (PBS). Two hours later, NC-5 (50 mg/kg/d, 75 mg/kg/d and 100 mg/kg/d) or oseltamivir phosphate (OS) (100 mg/kg/d) was given twice daily for 5 days through oral administration.

The lung index was calculated using the equation: lung index = (lung weight)/weight × 100. The lung injury score was evaluated according to the degree of congestion and edema of the lungs, the degree of bronchial necrosis, the degree of alveolar and bronchial epithelium injury, and the degree of inflammatory infiltration by a professional. In order to evaluate the infection in the mice, we homogenized the lungs of the mice, and filtered them using a 0.22 μm filter to evaluate the lung virus titer. The remaining processes were similar to the operation of measuring TCID50.

The related expression was observed in the animal experiments and was approved by the Science and Technology Department of Jiangsu Province (SYXK 2016-0011) and the Animal Ethics and Experimentation Committee of China Pharmaceutical University.

### 4.6. Western Blot Analysis

MDCK cells in 6-well plates were infected with 1.5 × 10^3^ TCID50 of the H1N1 influenza virus and treated with NC-5 (10 μM, 40 μM, and 80 μM) and OC (10 μM). Cells were lysed after 24 h. In a time-addition experiment, the MDCK cells were infected with virus at a multiplicity of infection (MOI) of 5, and co-incubated for 1 h at 4 °C. After changing the supernatant, the cells were cultured at 37 °C. NC-5 (80 μM) was added at distinct time-points, as described in Results. Ten hours post-infection, cell lysates were harvested. The following antibodies were applied for immunoblotting: anti-M1 (Catalog No. GTX125928, GeneTex), anti-NP (Catalog No. GTX83054, GeneTex), and anti-β-actin (Catalog No. SC-47778, Santa Cruz). A Bio-Imaging system was applied for band intensity detection, and the proteins were quantified using ImageJ, which was normalized to β-actin levels.

### 4.7. Luciferase Assay

The cell line 293T was used and cells were placed in a 24-well plate and then transfected with plasmids to express the influenza A virus A/WSN/33 (H1N1) PB1, PB2, PA, NP and vNS-Luc (150 ng of each) with pCMV β-gal (50 ng) for 6 h followed by the addition of NC-5 (10 μM, 40 μM and 80 μM), DMSO and ribavirin (RBV) (40 μM). The cell lysates were collected after 24 h and subjected to the luciferase assay [22].

### 4.8. Real-Time qPCR Analysis

MDCK cells were placed in 6-well plates and infected with 1.5 × 10^3^ TCID50 of the H1N1 influenza virus for 2 h followed by the addition of 40 μM NC-5. Cells were harvested at 6 h post-infection [27], and total RNA was extracted as the template. Reverse-transcription was performed using the PrimeScript TM Reagent Kit (Takara), and qPCR amplification was performed with a 7300 qPCR-PCR System (ABI) using SYBR Green and GAPDH as the endogenous control. Three independent experiments were performed. The 2^−△△*C*t^ method was used to calculate the relative RNA expression of NP and M1.

The primers used to detect the viral mRNAs were as follows:

NP forward primer: 5′-CCCAGGATGTGCTCTCTGAT-3′

NP reverse primer: 5′- TGAAAGGGTCTATTCCGACT-3′

M1 forward primer: 5′-TTCTAACCGAGGTCGAAAC-3′

M1 reverse primer: 5′-AAGCGTCTACGCTGCAGTCC-3′

GAPDH forward primer: 5′-CACTCACGGCAAATTCAACGGCAC-3′

GAPDH reverse primer: 5′-GACTCCACGACATACTCAGCAC-3′

### 4.9. Fluorescence Microscopy

MDCK cells were plated in a 6-well plate and infected with 1.5 × 10^3^ TCID50 of the H1N1 influenza virus for 2 h then treated with NC-5 (10 μM, 40 μM and 80 μM), DMSO and 10 μM OC for 24 h. After that, the cells were fixed with 4% paraformaldehyde and PBS containing 0.5% saponin was used to permeabilize the cells. Subsequently, the cells were stained with primary and fluorescently labeled secondary antibodies. The nuclei were stained with 4-6-diamidino-2-phenylindole (DAPI). Cells were observed using a fluorescence microscope.

### 4.10. Neuraminidase (NA) Inhibition Assay

NA enzyme inhibition assays were performed as previously described [28]. Influenza viruses H1N1, H1N1-H275Y, and H3N2 or CHO-expressed viral NAs H5N1, H7N9 and H7N9-R294K were incubated with NC-5 at different concentrations at 37 °C for 10 min. Then, 20 μM of fluorescent substrate 2′-(4-Methylumbelliferyl)-α-D-*N*-acetylneuraminic acid (MUNANA; Sigma) was added and incubated at 37 °C for 60 min. The fluorescence was measured with an AFLx 800 fluorescence microplate reader, and the excitation wavelength and emission wavelength were set to 360 nm and 450 nm, respectively.

### 4.11. Data Analysis

All experiments were performed three times. For survival studies, Kaplan–Meier survival curves were generated and compared by the log-rank test. Data were analyzed by one-way analysis of variance. The results are presented as the mean ± S.D. Unpaired two-tailed Student’s t test was used to determine the differences between the control and treated groups with significance set at *p* <0.05.

## 5. Conclusions

In this paper, we explored the anti-influenza activity of the diethyl triazole benzoic acid derivative, NC-5. This compound can inhibit influenza A viruses A/FM/1/47 (H1N1), A/Beijing/32/92 (H3N2) and oseltamivir-resistant mutant A/FM/1/47-H275Y (H1N1-H275Y) in a dose-dependent manner. Furthermore, in the cytopathic effect assay, it showed a 50% cytotoxic concentration (CC_50_) over 640 μM. Moreover, it can provide a great protective effect against wild-type and oseltamivir-resistant virus-infected mice. In summary, NC-5 possesses potent antiviral activity including towards oseltamivir-resistant mutants both in vitro and in vivo, which means that it presumes broad-spectrum antiviral activity. Therefore, it can be regarded as a promising candidate for influenza virus infection therapy. Its modification to improve the efficacy, further mechanistic studies and additional preclinical development will form part of our future work.

## Figures and Tables

**Table 1:** Structures and influenza A virus inhibition rates of newly synthesized compounds at concentrations of 10 μM and 100 μM.

Compound	R^a^	Inhibition Rate (%)	
		A/FM/1/47 (H1N1)	
		10 μM	100 μM
1	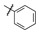	7.3 ± 4.66	7.8 ± 3.2
2	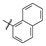	16.0 ± 6.14	30.3 ± 6.71
3	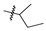	8.5 ± 3.50	16.8 ± 4.05
4	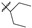	12.8 ± 4.88	23.6 ± 8.92
5 (NC-5)	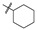	16.7 ± 5.76	83.7 ± 7.38
OC ^b^		55.2 ± 6.89	ND ^c^

Inhibition rate (%) = [(mean optical density of test—mean optical density of virus controls)/. (mean optical density of cell controls—mean optical density of virus controls)] × 100. The results are presented as the mean ± standard deviation (S.D.) of three independent cytopathic effect (CPE) reduction assays. ^a^ substituent group on the triazole. ^b^ oseltamivir carboxylate. ^c^ not detected.

## Abbreviations

NC-5	4-[2,2-bis(hydroxymethyl)-5-oxopyrrolidin-l-yl]-3-(5-cyclohexyl-4H-1, 2,4-triazol-3-yl) amino) benzoic acid
NA	neuraminidase
H1N1	influenza A virus A/FM/1/47 (H1N1)
H1N1-H275Y	influenza A virus A/FM/1/47-H275Y (H1N1-H275Y)
H3N2	influenza A virus A/Beijing/32/92 (H3N2)
H5N1	influenza A virus A/Vietnam/1203/2004 (H5N1)
H7N9	influenza A virus A/Anhui/1/2013 (H7N9)
H7N9-R294K	influenza A virus A/Anhui/1/2013-R294K (H7N9-R294K
MDCK	Madin–Darby canine kidney cells
DMEM	Dulbecco’s modified Eagle’s medium
CHO-K1	Chinese hamster ovary
OS	oseltamivir phosphate
OC	oseltamivir carboxylate
RBV	ribavirin
CPE	cytopathic effect
EC50	50% effective concentrations
CC50	50% cytotoxic concentration
TCID50	50% tissue culture infectious dose
H & E stain	haematoxylin and eosin stain
